# Recent advances in iNKT cell development

**DOI:** 10.12688/f1000research.21378.1

**Published:** 2020-02-20

**Authors:** Kristin Hogquist, Hristo Georgiev

**Affiliations:** 1 Center for Immunology, University of Minnesota, Minneapolis, MN, 55455, USA

**Keywords:** invariant natural killer T cells, subsets, development, T cell receptor signalling, thymus, CD1d, lipid, thymus, agonist selection

## Abstract

Recent studies suggest that murine invariant natural killer T (iNKT) cell development culminates in three terminally differentiated iNKT cell subsets denoted as NKT1, 2, and 17 cells. Although these studies corroborate the significance of the subset division model, less is known about the factors driving subset commitment in iNKT cell progenitors. In this review, we discuss the latest findings in iNKT cell development, focusing in particular on how T-cell receptor signal strength steers iNKT cell progenitors toward specific subsets and how early progenitor cells can be identified. In addition, we will discuss the essential factors for their sustenance and functionality. A picture is emerging wherein the majority of thymic iNKT cells are mature effector cells retained in the organ rather than developing precursors.

## Introduction

Identified by their T-cell receptor (TCR) specificity for lipids, invariant natural killer T (iNKT) cells are innate-like αβ T cells capable of releasing cytokines almost instantly upon stimulation without the need for prior activation ^1, 2^. Like conventional αβ T cells, iNKT cells arise from common lymphoid progenitors and run through their developmental program in the thymus. At the double-positive (DP) stage, their developmental programs bifurcate: While conventional αβ T cells get positively and negatively selected by thymic epithelial cells presenting peptide antigens by classical class I and II major histocompatibility complex (MHC) molecules ^3, 4^, iNKT cell progenitors are selected by other DP thymocytes presenting lipid antigens by CD1d, a non-classical MHC-like molecule ^5– 8^. Strong TCR signaling is required at this stage (referred to as agonist selection) ^9^ for upregulation of Egr2 ^10, 11^ and PLZF ^12, 13^, the latter of which is a master regulator of iNKT cell development. This consequently commits the DP αβ T-cell progenitor with the “right” TCR rearrangement to the iNKT cell pathway ^14, 15^. In addition to the strong TCR stimulation, auxiliary co-stimulatory signals are required by engaging CD80/CD86 ^16^ and via homotypic interactions between signaling lymphocyte activation molecule family (SLAMF) receptors, Slamf1 and Slamf6 ^17^. Following selection, iNKT cells complete their developmental program in the thymus and can egress to peripheral tissues. However, a substantial number are retained in the thymus, ending up as terminally differentiated functional subsets in this organ.

Despite the latest insights in the field of iNKT cell biology, the development of iNKT cell subsets and their differentiation pathways remain puzzling ^14, 15, 18– 21^. In this review, we will consider the contemporary understanding of iNKT cell subset development and in parallel we will discuss factors required for their maintenance and proper function. Moreover, we will focus on TCR signal strength involvement in iNKT cell lineage commitment and stability.

## The developmental map of iNKT cells

**Figure 1.**
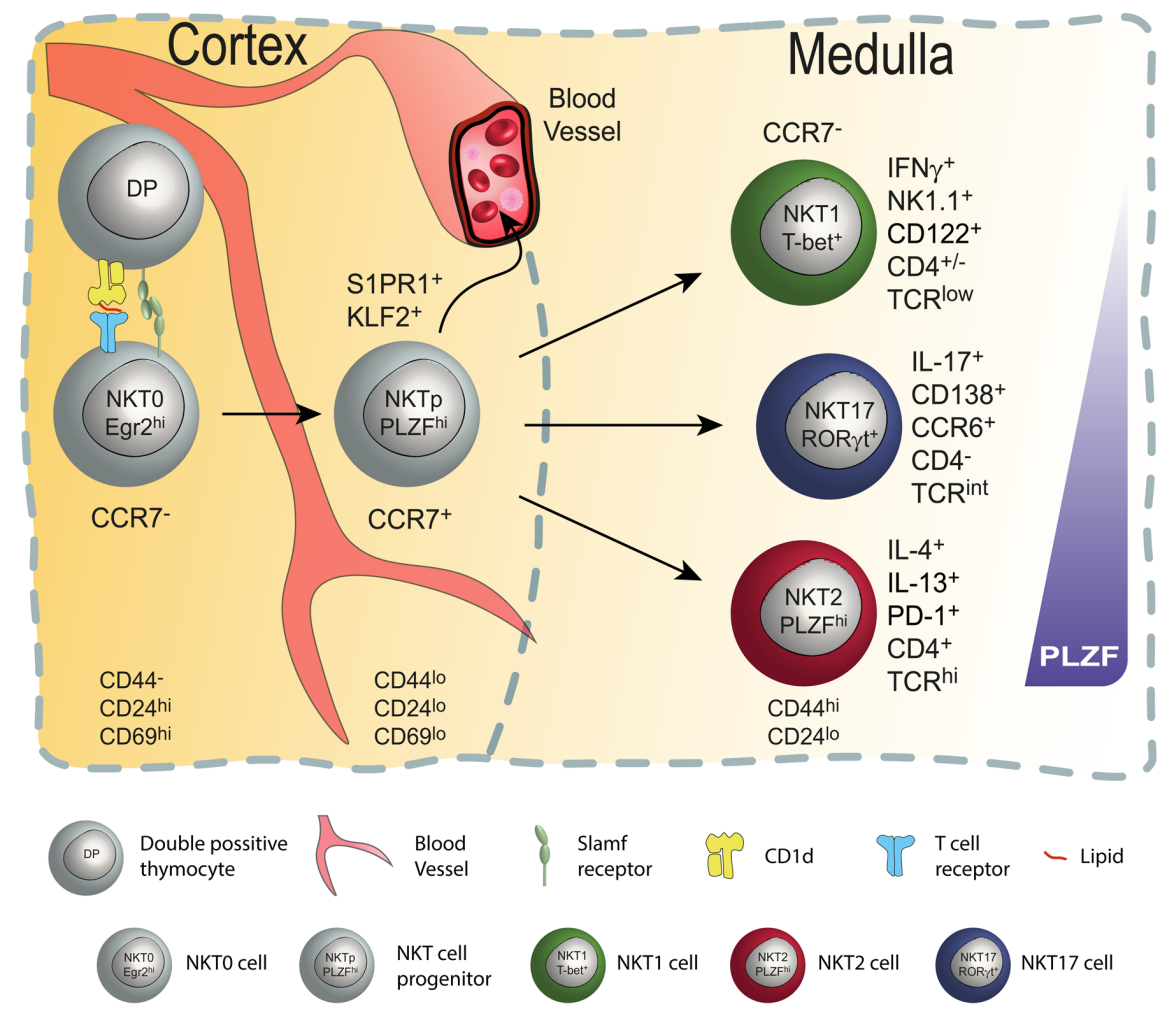
Invariant natural killer T (iNKT) cell development in the thymus. In the cortex of the thymus (left), double-positive (DP) iNKT progenitors are positively selected by other DP thymocytes presenting lipids via CD1d. This results in survival and lineage commitment; only those rare DP thymocytes bearing T-cell receptors (TCRs) (invariant Vβ14 chain paired with a limited set of beta chains) with the right specificity are selected and committed to the iNKT cell lineage. This step requires strong TCR signaling in combination with co-stimulatory signals via homotypic interactions between SLAM (signaling lymphocyte activation molecule) family members. Signaling leads to upregulation of Egr1 and Egr2, which are needed for PLZF induction and stable expression. Immediate post-selection iNKT cells are Egr2 ^hi^CD24 ^hi^CD44 ^−^CD69 ^hi^ and are designated as NKT0 cells. Subsequently, NKT0 cells downregulate CD24 and transition into CCR7 ^+^ multi-potent NKT cell progenitors (NKTp). At that stage, NKTp cells can egress from the thymus or continue their differentiation into one of the effector subsets (iNKT1, 2, or 17). Mature NKT cells are CCR7 ^−^ and reside in the medulla region as terminally differentiated tissue-resident NKT cell subsets.

The initial studies investigating iNKT cell development postulated that all iNKT cells execute the same developmental program divided into four stages (S0–S3). According to this model, iNKT cells progress from the most immature stage S0 (CD24 ^+^CD44 ^−^NK1.1 ^−^) to their final mature stage S3 (CD24 ^−^CD44 ^+^NK1.1 ^+^) by losing CD24 expression and subsequently upregulating first CD44 (in stage S2) and lastly natural killer NK1.1 (in stage S3) ^22, 23^. Although this holds true for some iNKT cells, the latest data demonstrate that this model does not apply to all iNKT cells. For instance, this model fails to incorporate interleukin-17 (IL-17)-producing iNKT cells ^24– 26^, it does not account for iNKT cells that produce high levels of IL-4 but never express NK1.1, and it cannot be employed with mouse strains that do not express NK1.1 ^27^. Therefore, a new functional classification of iNKT cells into three terminally differentiated subsets, which is based on the expression pattern of characteristic cytokines and transcription factors, was proposed ^28, 29^. In this model, all iNKT cells arise from a common progenitor designated as NKT0 cells (Egr2 ^hi^CD24 ^+^) and further differentiate into NKT1, NKT2, or NKT17 cell subsets. NKT1 cells (PLZF ^lo^Tbet ^+^) produce interferon gamma (IFNγ) and low levels of IL-4 upon stimulation. In addition, they are the only subset expressing NK cell signature proteins like NK1.1, NKG2D, Nkp46, and a cytotoxic gene expression program ^30– 32^. NKT2 cells express the highest levels of PLZF and IL-4. Lastly, NKT17 (PLZF ^int^RORγt ^+^) cells produce IL-17. Of note, only NKT2 cells are shown to actively produce and secrete IL-4 under steady-state conditions, an essential process for CD8 innate-like T-cell generation in the thymus and periphery ^28, 33– 38^. In this model, NKT1 cells, IL-4–producing NKT2 cells, and NKT17 cells are considered terminally differentiated cells which generally do not give rise to any of the other subsets ^24, 25, 28^. Subsequently, three independent groups performed transcriptome analysis of thymic-derived iNKT cell subsets and congruently observed distinct gene expression patterns for each subset ^30– 32^. Only NKT1 cells pass through all the stages of development S0–S3 defined by the original model. In contrast, NKT2 and NKT17 cells finish their maturation as terminally differentiated effector cells at stage 2. Taken together, these data widely validate the foundations of the NKT1/2/17 concept ( Figure 1).

Two additional subsets—NKT10 and NKT follicular helper (NKT _FH_) cells—have recently been proposed as an extension to the iNKT cell subset family; IL-10–producing E4BP4 ^+^ NKT10 cells were resident in the adipose tissue ^39– 41^, and Bcl-6 ^+^IL-21 ^+^ NKT _FH_ cells were found in germinal centers ^42, 43^. Notably, these two functional subsets have been described only in the periphery and are not present in the thymus.

## The complexity of iNKT cell subsets

Although current data indicate that NKT1, 2, and 17 cells are terminally differentiated functional iNKT cell subsets, the latest data bring a further level of complexity and reflect advances in the field. For instance, NKT1 cells segregate into CD4 ^+^ and CD4 ^−^ fractions. CD4 ^−^ NKT1 cells were shown to display a more NK-like phenotype with preferential expression of NK cell signature receptors and soluble cytotoxic mediators (for example, granzyme a, b, and perforin) ^30^. In contrast, CD4 ^+^ NKT1 cells express higher levels of NK cell–unrelated genes like IL-4 and CD81 ^30, 44^. In light of these findings, new questions arise about iNKT cell subset development and function. For example, are both NKT1 fractions distinct terminally differentiated cell subsets with divergent functions or do they represent intermediate versus fully matured stages of the NKT1 cell subset? Along the same line, a recent study described an alternative iNKT cell developmental pathway where a small CD4 ^−^ NKT1 population can arise from double-negative (DN) stage thymocytes ^45^. However, this pathway seems to contribute in only a minor way to the mature CD4 ^−^ NKT1 cell pool.

Even though less is known about NKT17 cell development ^46^, a recent study provided valuable insight into NKT17 biology ^47^. By evaluating the expression pattern of the NKT17 characteristic genes CD138 ^48^ and CCR6 ^26^, the investigators suggested the final steps of the NKT17 developmental pathway from RORγt ^+^ NKT17 committed progenitors which progressively gain CD138 followed by CCR6 to become CD138 ^+^CCR6 ^+^ DP mature NKT17 cells ^47^.

Expectedly, the PLZF ^hi^ iNKT cells display the highest level of heterogeneity ^28, 30^ since this cell fraction encompasses both mature NKT2 and immature NKT progenitor cells ^28, 49^ ( Figure 1). Yet, in a 2016 study, Engel *et al*. performed a single-cell RNA-sequencing analysis on each of the iNKT cell subsets from thymic origin ^31^. In addition to including NKT1, 2, and 17 cells, this study included the most immature NKT0 cells which are still CD24 ^hi^ and have recently undergone selection. Despite that, authors still detected some NKT2 cells with transcripts of characteristic genes known to be highly expressed in recently selected cells (for example, *Itm2a*, *Ccr9*, and *Ldhb*) ^31, 50, 51^. In line with that, principal component analysis of NKT0, 1, 2, and 17 cells showed that each of these fractions segregated as different subsets with only marginal overlap between NKT0 and NKT2 cells ^31, 32^. Taken together, these data argue for a missing link in the development of the terminally differentiated iNKT cell subsets from the CD24 ^hi^ NKT0 stage. As mentioned above, there are several reported genes with a shared expression pattern between NKT0 and some CD24 ^−^PLZF ^hi^ cells. Hence, they might be suitable as markers for iNKT cell subset progenitors “hiding” within the PLZF ^hi^ iNKT cell fraction. Such a candidate is CCR7, which was shown to be expressed on both cell fractions at RNA ^30, 31^ and protein ^30, 52^ levels. Indeed, a recent study by Wang *et al*. described CCR7 as a characteristic marker for multi-potent iNKT cell progenitors (NKTp) ^52^ ( Figure 1). In that study, the authors took advantage of the KN2 mouse model in which IL-4–secreting cells can be identified by human CD2 (hCD2) expression ^53^. Those experiments demonstrated that the small fraction of CCR7 ^+^ iNKT cells did not produce IL-4 yet could give rise to all iNKT effector subsets in the thymus and periphery.

## CCR7 ^+^ cells (amongst NKT2) represent an undifferentiated precursor that emigrates from the thymus

Although previous studies had shown first that NK1.1 ^−^ iNKT ^22, 23^ cells and later that “NKT2-like” cells ^30^ can emigrate into the periphery, these reports did not investigate CCR7 expression on recent thymic emigrants (RTEs). Interestingly, Wang *et al*. also found that CCR7 ^+^ iNKT cells, despite representing only about 5% of total thymic iNKT cells, were prominent amongst RTEs, a process stringently dependent on Klf2 and S1PR1 ^52^ ( Figure 1). This suggests that undifferentiated NKTp cells exit the thymus and can initiate or complete (or both) differentiation in peripheral tissues. Indeed, 5 days after adoptive transfer, CCR7 ^+^ NKTp cells differentiated into all three effector subsets whether they had been transferred directly into the thymus or intravenously into the periphery. Moreover, parabiosis experiments showed that more than 99% of all thymic iNKT cells were tissue-resident as opposed to arriving from circulation ^52^. Thus, only a small fraction of “developing” NKTp cells in the thymus are constantly replenishing the pool of iNKT cells in the thymus and periphery.

Overall, these data raise the logical questions: What are the precise signals specifying iNKT cell differentiation, and what are the checkpoints for subset commitment? Moreover, does commitment occur at the DP stage in the cortex or at a later developmental stage post-selection as the undifferentiated CCR7 ^+^ cell encounters a new cellular milieu in the thymic medulla? In fact, recent evidence suggests a combination of the two. These new findings are discussed in the following section.

## TCR signaling: strength and context

**Figure 2.**
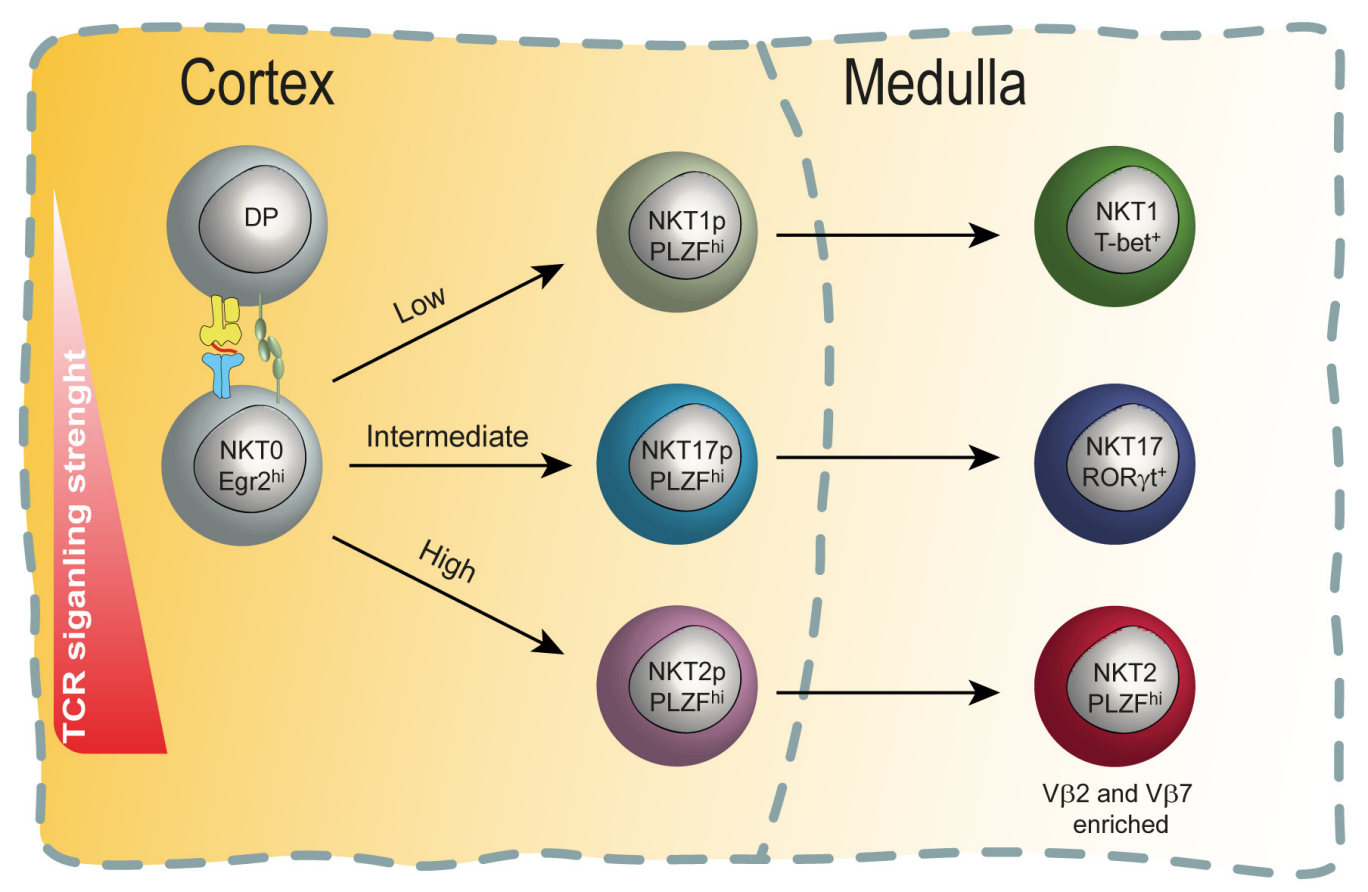
Model of T-cell receptor (TCR) signaling in the cortex for invariant natural killer T (iNKT) differentiation. In this model, TCR binding strength (avidity) during the cortical selection process steers stage 0 iNKT cells to specific cell fates. For iNKT cells, which express a semi-invariant TCR alpha chain, TCR beta chain usage can influence TCR binding avidity. For example, Vβ2 and Vβ7 chains tend to confer higher binding avidity. TCRs with high avidity drive NKT2 differentiation preferentially, TCRs with intermediate binding avidity drive NKT17 differentiation, and those with low binding avidity drive commitment to the NKT1 pathway. NKT progenitor (NKTp) cells finish their differentiation in the medulla.

It has long been known that TCR signaling is critical for iNKT cell development, as recognition of CD1d:lipid ligands is required for positive selection ^5– 8^. However, recent data suggest a critical role for TCR signaling in subset differentiation as well. Of interest in this regard is the observation that mature iNKT cell subsets exhibit different levels of TCR on their surface ^30, 54^ where NKT1 cells are low, NKT17 cells are intermediate, and NKT2 cells display the highest level of TCR expression. This also appears to correlate with their ongoing TCR signal strength under steady-state conditions ^54, 55^. Overall, this suggests a potential requirement for different TCR signal intensities in the development of each ( Figure 2). In fact, two independent groups recently addressed this question by exploiting the SKG mouse model, in which TCR signaling is weakened because of a hypo-morphic ZAP70 allele ^54, 56^. Hence, both studies showed that weakened TCR signaling led to abrogation in NKT2 and, to a lesser extent, NKT17 cell development while not reducing NKT1 cell development. In addition, genome-wide analysis of chromatin accessibility between NKT2 cells from SKG and wild-type control mice showed that gene regions coding MAPK/ERK and Notch pathway regulators were less accessible in the SKG mouse. Moreover, a recent study showed that a deficiency in TRAF3-interacting protein which facilitates MEK/ERK signaling at the trans-Golgi network led to a decrease in IL-4–producing NKT2 cells ^57^. This places TCR strength signaling as a possible modulator of MAPK/ERK and Notch signaling, which might influence iNKT cell subset development. Additionally, the Src homology2 domain-containing phosphatase 1 (Shp1)-deficient mouse showed an increase of NKT2 and NKT17 cells ^58^. Although the authors did not find direct evidence that this is due to altered TCR signaling, Shp1 was previously identified as a negative regulator of TCR signaling by targeting ZAP-70 ^59^.

One critical gene target of TCR signaling in iNKT cells is PLZF ^11^. Indeed, PLZF expression in iNKT subsets mirrors TCR levels and signaling; NKT2 expresses the highest level, NKT17 expresses intermediate, and NKT1 expresses the lowest level ^28^ ( Figure 1). An interesting recent study showed that, like that of TCR, the quantity of PLZF expressed by a given cell is a critical factor in iNKT subset differentiation. In that study, a hypomorphic allele of PLZF was found to strongly reduce NKT2 and NKT17 numbers while relatively sparing NKT1 cells ^60^. Collectively, these findings corroborate the idea that the quantity of TCR signaling is a critical factor in iNKT cell subset differentiation.

Since it is known that iNKT cell selection happens in the cortex ^61^, it seems likely that the interaction of stage 0 iNKT cells with CD1d/lipid-presenting DP thymocytes is the stage at which signal strength is critical ( Figure 2). In this regard, it is curious that cells immediately following the CCR7 ^+^ stage do not yet show signs of differentiation to distinct subsets and express a uniformly high level of TCR ^52^. Furthermore, the CCR7 ^+^ iNKT cell population has a transcriptome that does not yet resemble any of the differentiated subsets ^32^. Thus, it remains an open question whether CCR7 ^+^ cells are already “committed” to a specific cell subset or are multi-potent at the single-cell level. Future studies will need to address this by, for example, using single-cell adoptive transfer assays of cell fate or single-cell epigenomics analysis of CCR7 ^+^ cells ( Figure 2).

**Figure 3.**
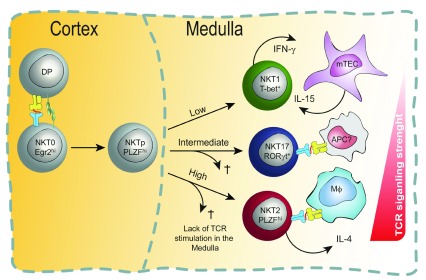
Model of T-cell receptor (TCR) signaling in the medulla for invariant natural killer T (iNKT) subset activation and survival. According to this non-mutually exclusive model, subset commitment decisions are made or reinforced (or both) in the thymic medulla. At the CCR7 ^+^ stage, developing natural killer cell progenitors (NKTp) may still be uncommitted to any particular subset (as shown). Alternatively, signaling at the double-positive (DP) stage may have altered their epigenomes such that they have a propensity to differentiate into one of the three major subsets (as depicted in Figure 2). Either way, following migration into the medulla, NKTp cells experience different strengths of TCR signaling—based on their TCR:CD1d/lipid binding avidity with distinct antigen-presenting cells (APCs) they stochastically encounter in the medulla—which governs their subset commitment or survival or both. Cells with the highest binding avidity survive as NKT2 cells, those with intermediate affinity survive as NKT17 cells, and only NKT1 cells survive without continued TCR stimulation. In the medulla, NKT2 cells require TCR–CD1d interaction with macrophages in order to produce interleukin-4 (IL-4) in the steady state, and NKT1 cells need IL-15 produced by medullary thymic epithelial cells (mTECs) for their proper differentiation and survival. It is unknown what cell types present lipids to NKT17 cells, although it is reported that they encounter intermediate TCR signaling in the steady state, which is crucial for their differentiation.

Another (non-mutually exclusive) possibility is that TCR signaling is critical during and following the CCR7 ^+^ stage ( Figure 3). CCR7 is a chemokine receptor that facilitates the movement of iNKT cells from the cortex to the medulla and is crucial for proper maturation and maintenance of resident iNKT cells in the thymus ^52^. It is possible that NKT cells require continued interaction with CD1d-expressing antigen-presenting cells in the medulla, particularly to maintain expression of survival factors, including bcl2 family members ^60^ ( Figure 3). Of note, medulla-derived factors have already been shown to play an essential role in iNKT cell subset homeostasis. For instance, it has been shown that IL-15 is required for terminal maturation and survival of NKT1 cells ^62^ and that IL-25 is implicated in NKT2 cell development and effector functions ^24, 63^. A new study by Wang *et al*. sought to discern whether steady-state IL-4 production by mature NKT2 cells is TCR–CD1d interaction-dependent ^55^. Indeed, this work showed that intrathymic transfer of NKT2 cells into CD1d-deficient recipient mice resulted in a loss of IL-4 production within 9 days after transfer ^55^. Moreover, using inducible knockout (KO) models, the authors identified the CD1d-presenting cell subset as macrophages. At this point, it is unknown whether the medullary macrophages that activate NKT2 cells present the same or distinct self-lipid ligands compared with the cortical DP thymocytes that initially select NKT cells ( Figure 2 and Figure 3).

Although NKT cells are often assumed to be mono-specific given the semi-invariant nature of their TCR alpha chain, evidence suggests that the particular TCR beta chain used can influence recognition ^64– 67^. In this context, it is interesting that the different NKT subsets have reproducible differences in TCR beta usage ^54^. Furthermore, retrogenic experiments showed that TCR:CD1d/lipid binding avidity positively correlated with selection efficiency. These avidity differences were conferred by different TCR beta chains. In addition, a longer half-life of binding favored the development of PLZF ^hi^ NKT2 cells over the other subsets ^68^. Further deep sequencing of the TCR alpha and beta repertoires of iNKT subsets could provide insight in the future.

As previously mentioned, SLAM family receptors (SFRs) play a crucial role in iNKT cell development ^17^. This family includes six members that can convey both activating or inhibitory signals depending on the adaptor proteins that are recruited upon receptor engagement ^69^. Lu *et al*. recently addressed the role of SLAM receptor proteins (SRPs) in iNKT cell development by generating a KO mouse strain for all six members of this family ^70^. They showed that loss of SFRs led to higher TCR signaling in developing iNKT cells, as judged by higher Nur77 and Egr2 levels, and to reduced numbers of all mature iNKT cells. In addition, they reported that the number of CD24 ^+^ immature iNKT cells was not altered. Considering these findings, the authors concluded that the initial positive selection of iNKT cells was unaffected in SFR-deficient mice but that the observed iNKT cell loss was due to augmented TCR signal strength driving apoptosis. The authors also conclude that inhibitory signals provided by SFRs attenuate TCR signal strength after positive selection to promote NKT cell development, as opposed to previous studies proposing that SFRs contributes with a positive signal that complements TCR signaling to support NKT cell development ^71^. Interestingly, however, Lu *et al*. provided evidence that Vβ usage was altered, whereby Vβ7 clones were preferentially enriched in SFR-deficient mice ^70^. Of note, it was reported that Vβ7 usage confers higher avidity binding to CD1d and is expressed mostly by NKT2 cells ^28, 54, 66^. As mentioned above, NKT2 cells are thought to experience the strongest TCR signaling in the steady state ^54, 55^. Hence, this raises the possibility that mainly clones with high-avidity Vβ chain rearrangements, which result in stronger TCR signaling, are able to survive in the absence of SFR co-stimulation. Strikingly, a previous study showed that in conditions of limited endogenous ligand concentrations, thymic selection favors Vβ7 ^+^ iNKT cells over iNKT cells expressing other Vβ chains ^66^. Thus, although Lu *et al*. show that iNKT cells from SFR-deficient mice have higher ongoing TCR signal strength in comparison with wild-type mice, this effect might be due to selective survival of high-avidity Vβ7-expressing NKT2 cells and not to lack of SFR-mediated inhibition. Therefore, further studies are needed to understand the precise role of SFR signaling in NKT cell development or subset differentiation or both.

## Concluding remarks

A couple of technical points regarding the development of iNKT cells can be gleaned from the studies we discussed here. First is that the paradigm of “staging” NKT cell development using CD44 and NK1.1 is relevant only to NKT1 lineage cells in B6 (C57BL/6) mice and thus should be employed with caution. Second, a high level of PLZF expression is not sufficient to distinguish functionally differentiated NKT2 cells from multi-potent NKTp cells. Therefore, CCR7, PD1, or other markers are needed to discriminate between developing and differentiating iNKT subsets. In addition, NKT2 cells were shown to actively produce and secrete high levels of IL-4 whereas multi-potent NKTp cells did not. Furthermore, most studies investigating cytokine production in iNKT cells use PMA/ionomycin as a stimulus, which might be misleading in this regard. Hence, reporter mouse models currently represent the best way to identify cells actively producing IL-4. In the future, it will be interesting and important to determine when NKT1 or NKT17 or both actively produce cytokine in uninfected animals.

Despite the recent progress in the field of iNKT cell biology, many questions remain unanswered. For instance, assuming surface TCR levels dictate TCR signaling quantity, what drives the high TCR expression on NKT2 and 17 cells? Is it a signal received during selection owing to specific Vβ chain usage or rather an environmental consequence such as high presence of endogenous cognate ligand? Moreover, what are the relevant self-lipid ligands in the thymus and do they differ between cortical and medullary antigen-presenting cells? How diverse are they and is this diversity detected? In this context, a recent study showed that non-agonist CD1d-associated lipids could alter lipid presentation and impact iNKT development in a complex way ^72^. Thus, many interesting questions remain open about the complex biology induced by the T-cell recognition of lipids.

## Abbreviations

DP, double-positive; iNKT, invariant natural killer T; MHC, major histocompatibility complex; NK, natural killer; NKT _FH_, invariant natural killer T follicular helper; NKTp, multi-potent invariant natural killer T-cell progenitor; RTE, recent thymic emigrant; SFR, SLAM family receptor; Shp1, Src homology2 domain-containing phosphatase 1
